# Branchial cleft cyst arising in posterior mediastinum: A case report

**DOI:** 10.3389/fsurg.2022.1088142

**Published:** 2023-01-06

**Authors:** Lin-Jie Li, Zhi-Feng Han, Sheng-Tao Shang

**Affiliations:** Department of Thoracic Surgery, China-Japan Union Hospital of Jilin University, Changchun, China

**Keywords:** branchial cleft cysts, posterior mediastinum, mediastinal cysts, diagnosis, treatment

## Abstract

Branchial cleft cysts are congenital diseases of the neck caused by abnormal embryonic development of the first to fourth branchial clefts. Most branchial cleft cysts are found in the head and neck, but branchial cleft cysts arising in posterior mediastinum are rarely reported. We report a 44-year-old Chinese man who was found to have a right-posterior mediastinal mass on chest computed tomography (CT) during a physical examination. The size of the mass was about 30.6 mm * 25.1 mm and enhanced CT of the chest showed an occupying lesion in the right parietal esophagus of the upper-posterior mediastinum with no significant enhancement. The patient was considered to have a neurogenic tumor with cystic change and underwent posterior mediastinal tumor resection. Postoperatively, pathological examination confirmed the final diagnosis of bronchial cleft cyst. The patient was discharged on the 7th day after surgery. One year postsurgery, no obvious recurrence was found in reexamination.

## Introduction

Clinically, the second branchial cleft cyst is more prevalent, accounting for approximately 90% of all branchial cleft cysts (1), followed by the first branchial cleft cyst. There are few clinical reports about the third and fourth branchial cleft cysts. Branchial cleft cysts normally occur in the neck, anterior to the sternocleidomastoid muscle (SCM), but rarely in the mediastinum, especially the posterior mediastinum. Branchial cleft cysts in the lower neck mostly occurred on the left side. The left dominance may be relevant to the deficiency or invagination of the right ultimobranchial body, furthermore, asymmetrical vascular defects may exists during the development of the fourth arch (2). The present case is a rare branchial cleft cyst located on the right side of the posterior mediastinum that has never been reported before.

## Case presentation

**Figure 1:**
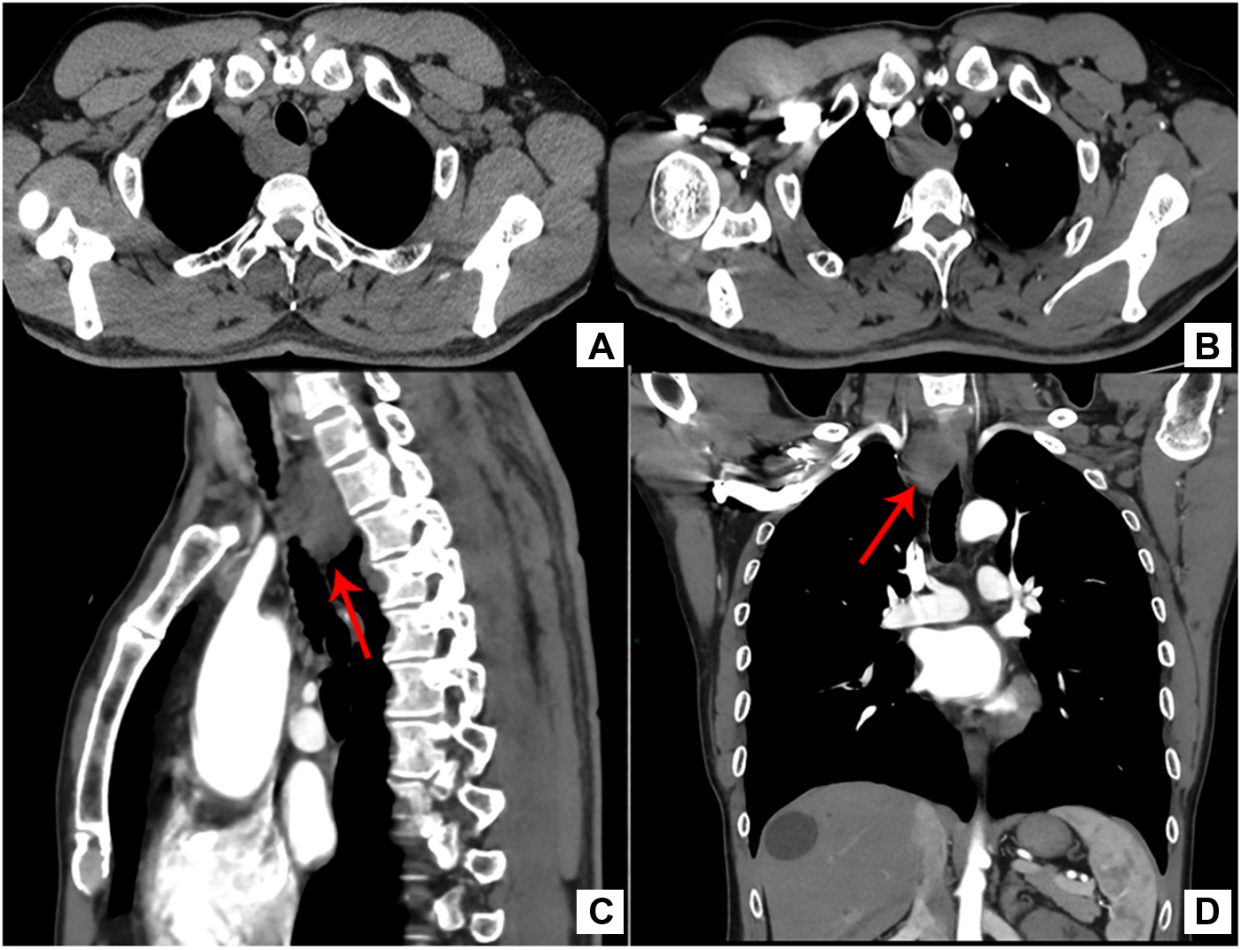
(**A**) CT revealed a mass on the right side of the upper and posterior mediastinum and the adjacent trachea and esophagus were pushed. ((**B**) Transverse, (**C**) sagittal, (**D**) Coronal) contrast-enhanced CT scan showed an unobvious enhanced mass with clear tumor boundaries.

A 44-year-old Chinese man was hospitalized due to a posterior mediastinal mass found on physical examination, without dyspnea, dysphagia, repeated fever and other discomforts. Chest CT showed that the right side of the upper and posterior mediastinum could be seen as a round and slightly low-density shadow with a clear boundary, the size of the lesion was about 30.6 mm * 25.1 mm, the CT value was about 27.0 HU, and the adjacent trachea and esophagus were pushed (Figure 1A). Contrast-enhanced CT showed an unobvious enhanced mass with clear tumor boundaries (Figure 1B–D). The preoperative diagnosis was neurogenic tumors with cystic changes based on the enhanced CT features.

**Figure 2:**
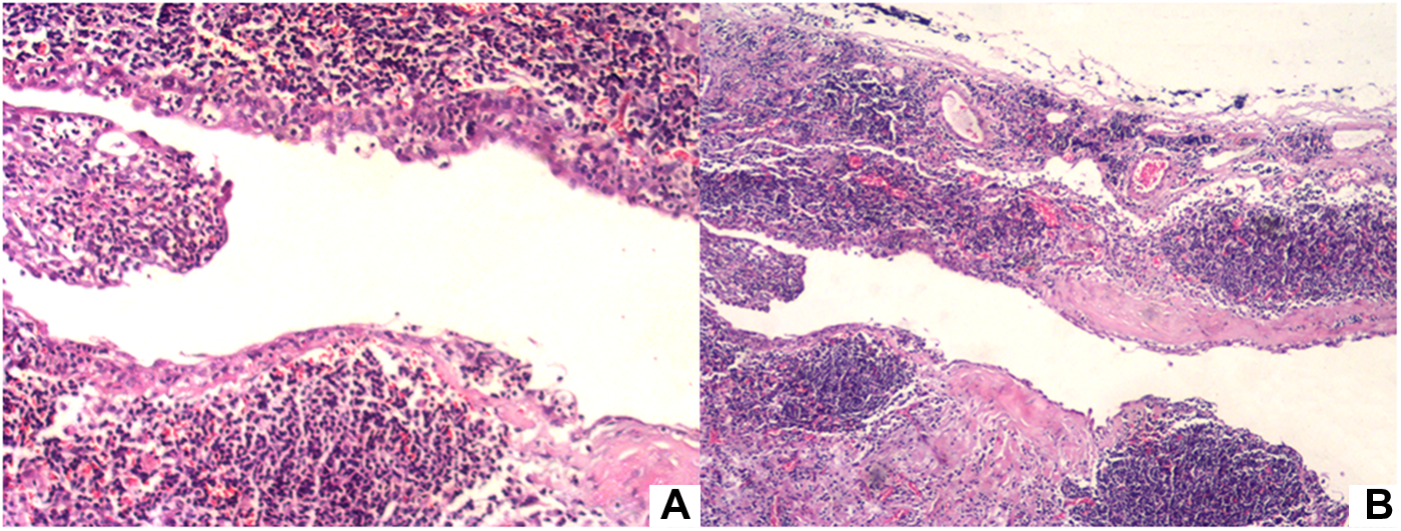
(**A**) Hematoxylin-eosin staining showed the fibrous cyst wall lined with squamous epithelium and cuboidal epithelium, with hyperplasia of lymphoid tissue and formation of lymphoid follicles.(×100) (**B**) ×40.

**Figure 3:**
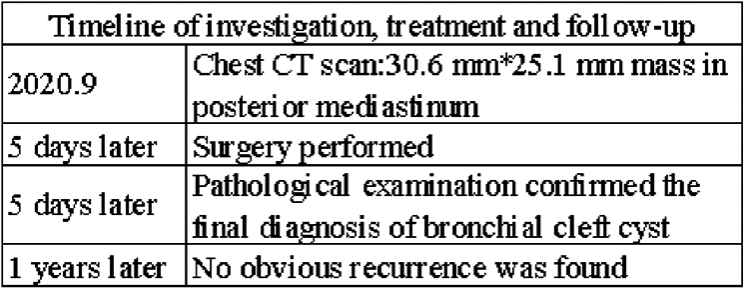
Timeline of investigation, treatment and follow-up.

The patient underwent posterior mediastinal cyst totally excise through video-assisted thoracoscope surgury (VATS) under general anesthesia. It was found that the lesion was located above the azygos arch in the superior and posterior mediastinum, on the right side of the esophagus, and protruded from the surface of the mediastinal pleura, with a size of about 30 mm * 30 mm. Intraoperatively, the cyst had a smooth surface, fixed position, clear boundaries and no invasion of surrounding tissues. The cyst was completely excised. The patient recovery and was discharged on the 7th day after surgery. Postoperative pathology examination showed the fibrous cyst wall lined with squamous epithelium and cuboidal epithelium, with hyperplasia of lymphoid tissue and formation of lymphoid follicles, consistent with branchial cysts (Figures 2A,B). One year after surgery, no significant recurrence was found when the patient was reexamined (Timeline in Figure 3).

## Discussion

In 1832, Ascherson firstly suggested that branchial cysts were the result of imperfect obliteration of a pharyngeal cleft (3). In the time of the fourth week of embryonic life, the branchial organ, composed of six pairs of mesodermic arcs, arises surrounding the developing pharynx. If the fissure or part of the cystic gland does not fully invaginate before the seventh week of embryonic life, the trapped remainder will form an intra-epithelial cyst (4). There are various theories about the origin of branchial cleft cysts: degenerative cystic changes of cervical lymph nodes, hypopharyngeal duct theory, parotid gland inclusion theory (5), However, the currently most accepted theory is that branchial cleft cysts are caused by persistent degenerated abnormal branchial organs located on the lateral side of the neck.

The most common clinical presentation in patients with bronchial cleft cysts is a non-painful neck lump on the left side along the anterior border of the SCM (6). Similar to other mediastinal masses, branchial cleft cysts in the mediastinum can produce the same compression symptoms, such as pushing the esophagus and trachea causing dysphagia or respiratory distress.In William's study,he considered that all the mediastinal branchial cleft cysts started from the fourth branchial cleft (7). Therefore, the most common clinical manifestations of fourth branchial cleft cysts, namely recurrent neck abscesses and acute suppurative thyroiditis, can also be observed (8). Diagnosis of branchial cleft cysts relies on pathological examination. Fine-needle aspiration (FNA) cytology is useful for preoperative pathological diagnosis and it can help exclude metastatic or inflammatory disease, which will effectively avoid unnecessary surgery. The cytological criteria for diagnosing branchial cleft cysts are the presence of nuclear keratinocytes and squamous epithelial cells of varying degrees of maturity, as well as lymphoid aggregates in subepithelial lymphoid tissue structures (6). Ultrasonography (USG) is widely used as an inexpensive and convenient examination that can characterize cysts and guide FNA procedures. CT scans and magnetic resonance imaging (MRI) can be used to show the nature of the cyst and the anatomical relationship of the lesion. In addition, barium meal and laryngoscopy play a considerable part in judging the location of the fistula and guiding the scope of surgical resection. Surgery is the primary therapy for branchial cleft cysts, and complete resection of the lesions can reduce the probability of postoperative recurrence. For branchial cleft cysts in the neck, a broad horizontally oriented incision is essential to fully expose the lesion and to avoid intraoperative injury, and inappropriate surgical maneuvers are likely to lead to recurrence. For branchial cleft cysts in the mediastinum, video-assisted thoracoscopic surgery is recommended, and its safety and efficacy have been well documented in previous studies. Other treatment approaches include injection of sclerotic agents and endoscopic cauterization.Surgical treatment is not recommended in the case of acute infection or abscess (6, 8).

Mediastinal cysts comprise 12 to 20 percent of the total mediastinal masses and foregut cysts are the most general occurring mediastinal cysts, including bronchogenic cysts and esophageal cysts (9). It's typically detected bronchial cysts near the tracheal neck in the middle or posterior mediastinum. Bronchogenic cysts present as single, clear-bounded round masses without enhancement on CT (10). Foregut cysts also contain another specific type, the neurenteric cysts, which generally have a fibrous junction in the vertebral column or an intra-spinal component. CT and MRI show a thin-walled paravertebral cystic mass with vertebral abnormalities and intraspinal elongation of the cyst (11). The appearance of branchial cleft cysts on CT makes it difficult to distinguish them from bronchogenic cysts on imaging. Therefore, pathological testing is ultimately required for identification. Bronchial cysts are filled with pseudolaminate columnar respiratory epithelia and contain bronchial glands and cartilage (9).

The preoperative diagnosis of our patients was neurogenic tumor with cystic change. Neurogenic tumors are the most representative of posterior mediastinal tumors. Cystic changes are more common in schwannomas than in other neurogenic tumors. Schwannoma presents on CT as a clearly defined round or oval mass. Areas of heterogeneity correlate with cystic lesions, hemorrhagic lesions, or cellular reduction. Punctate, mottled, curvilinear calcifications can be observed along the walls of schwannomas (11). Other common neurogenic tumors with cystic changes include neurofibromas and ganglioneuromas.

## Conclusion

Branchial cleft cyst is a congenital disease of the neck caused by abnormal degeneration of branchial organs, which occurs very rarely in the posterior mediastinum. When cystic lesions of the posterior mediastinum are found, bronchial cleft cysts are one of the noteworthy differential diagnoses. Radiological examinations (CT and MRI), ultrasound-guided FNA may assist in preoperative diagnosis. Surgery is the most effective method for the treatment of this disease.

## Data Availability

The original contributions presented in the study are included in the article/Supplementary Material, further inquiries can be directed to the corresponding author/s.

## References

[1] Lee DH Yoon TM Lee JK Lim SC. Clinical study of second branchial cleft anomalies. J Craniofac Surg. (2018) 29(6):e557–60. 10.1097/SCS.0000000000004540 29608472

[2] Cain RB Kasznica P Brundage WJ. Right-sided pyriform sinus fistula: a case report and review of the literature. Case Rep Otolaryngol. (2012) 2012:934968. 10.1155/2012/934968 22953130 PMC3420472

[3] Ascherson FM. De fistulis colli congenitis, adiecta fissurarum branchialium in mammalibus avibusque historia succincta: Commentatio pro venia docendi. C. H. Jonas, Berolini (1832). p. 1–21.

[4] Houck J. Excision of branchial cysts. Oper Tech Otolaryngol Head Neck Surg. (2005) 16:213–22. 10.1016/j.otot.2005.09.007

[5] Bhaskar SN Bernier JL. Histogenesis of branchial cysts; a report of 468 cases. Am J Pathol. (1959) 35:407–43. PMCID: PMC1934860 13627135 PMC1934860

[6] Zaifullah S Yunus MR See GB. Diagnosis and treatment of branchial cleft anomalies in UKMMC: a 10-year retrospective study.’ European archives of oto-rhino-laryngology. Eur Arch Otorhinolaryngol. (2013) 270:1501–6. 10.1007/s00405-012-2200-7 23053382

[7] Downey WL Ward PH. Branchial cleft cysts in the mediastinum. Arch Otolaryngol, Chicago, Ill. (1969) 89:762–5. 10.1001/archotol.1969.00770020764017 5778138

[8] Nicoucar K Giger R Pope HG Jaecklin T Dulguerov P. Management of congenital fourth branchial arch anomalies: a review and analysis of published cases. J Pediatr Surg. (2009) 44:1432–9. 10.1016/j.jpedsurg.2008.12.001 19573674

[9] Duwe BV Sterman DH Musani AI. Tumors of the mediastinum. Chest. (2005) 128:2893–909. 10.1378/chest.128.4.2893 16236967

[10] Ozawa Y Hiroshima M Maki H Hara M Shibamoto Y. Imaging findings of lesions in the middle and posterior mediastinum. Jpn J Radiol. (2021) 39:15–31. 10.1007/s11604-020-01025-0 32740793

[11] Nakazono T Yamaguchi K Egashira R Takase Y Nojiri J Mizuguchi M CT-based mediastinal compartment classifications and differential diagnosis of mediastinal tumors. Jpn J Radiol. (2019) 37:117–34. 10.1007/s11604-018-0777-5 30238278

